# Recent Advances in Silent Gene Cluster Activation in *Streptomyces*

**DOI:** 10.3389/fbioe.2021.632230

**Published:** 2021-02-18

**Authors:** Zhenyu Liu, Yatong Zhao, Chaoqun Huang, Yunzi Luo

**Affiliations:** ^1^Key Laboratory of Systems Bioengineering (Ministry of Education), Frontier Science Center for Synthetic Biology, School of Chemical Engineering and Technology, Tianjin University, Tianjin, China; ^2^Collaborative Innovation Center of Chemical Science and Engineering (Tianjin), Tianjin University, Tianjin, China

**Keywords:** *Streptomyces*, natural products, biosynthetic gene cluster, heterologous expression, *in situ* activation, synthetic biology

## Abstract

Natural products (NPs) are critical sources of drug molecules for decades. About two-thirds of natural antibiotics are produced by *Streptomyces. Streptomyces* have a large number of secondary metabolite biosynthetic gene clusters (SM-BGCs) that may encode NPs. However, most of these BGCs are silent under standard laboratory conditions. Hence, activation of these silent BGCs is essential to current natural products discovery research. In this review, we described the commonly used strategies for silent BGC activation in *Streptomyces* from two aspects. One focused on the strategies applied in heterologous host, including methods to clone and reconstruct BGCs along with advances in chassis engineering; the other focused on methods applied in native host which includes engineering of promoters, regulatory factors, and ribosomes. With the metabolic network being elucidated more comprehensively and methods optimized more high-thoroughly, the discovery of NPs will be greatly accelerated.

## Introduction

Natural products (NPs) are major sources of drug molecules, including antibiotic, anticancer, antifungal, antiparasitic, and immunosuppressive compounds. *Streptomyces* plays a central role in the discovery of NPs, and the genes responsible for NPs biosynthesis are generally clustered in a continuous region of the genome termed as biosynthetic gene clusters (BGCs). With the rapid development of sequencing technologies, especially the third generation sequencing technology (Loman and Pallen, 2015), more and more genomic information of *Streptomyces* was clarified. Analysis of sequenced *Streptomyces* genome data revealed that a single *Streptomyces*’ genome generally encodes 25–50 BGCs, ∼90% of which are silent or cryptic under standard laboratory growth conditions (Walsh and Fischbach, 2010; Rutledge and Challis, 2015; Mao et al., 2018). Therefore, to increase the production of the encoded natural product, methods to unlock or up-regulate these so called “silent” gene clusters have become the interest of research in recent years.

**FIGURE 1:**
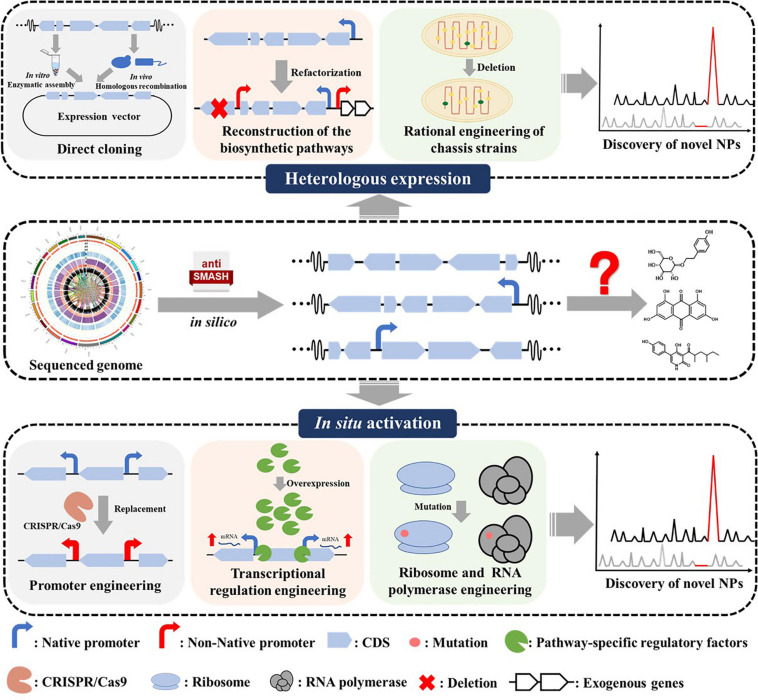
Overview of strategies for silent BGCs activation. BGC, biosynthetic gene cluster; CDS, coding sequences; RBS, ribosome binding site.

Numbers of methods have been developed to activate silent BGCs in recent years (Rutledge and Challis, 2015; Onaka, 2017; Mao et al., 2018; Lewis, 2020). Powerful bioinformatics approaches for genome mining and identification of NPs BGCs are well summarized in some recent reviews (Lee et al., 2020; Ren et al., 2020; Van Santen et al., 2020; Kenshole et al., 2021). Herein, we provide a concise overview as an introductory guide to the recent advances in silent BGCs activation in *Streptomyces* from two aspects, involving heterologous reconstruction and *in situ* activation (Figure 1). For heterologous reconstruction, we discussed different cloning strategies, biosynthetic pathways reconstruction methods, and chassis strain engineering approaches. For *in situ* activation, we summarized the methods including promoter engineering, transcription factors operating, and ribosome engineering (Table 1).

**TABLE 1:** Examples of BGCs activation in *Streptomyces.*

**Hosts**	**Targets**	**Approaches**	**Expression strains**	**Effect**	**References**
Heterologous host	Cloning BGC	Environmental DNA (eDNA) cosmid libraries	*S. albus* J1074	To clone the complete malacidin BGC from environmental metagenome samples	Hover et al., 2018
		*Streptomyces* bacterial artificial chromosome system (pSBAC)	*S. lividans and S. coelicolor*	To clone a 60 kb pikromycin BGC	Pyeon et al., 2017
		Exonuclease Combined with RecET recombination (ExoCET)		To clone a 106 kb salinomycin BGC	Wang et al., 2018
		Cas9-Assisted Targeting of chromosome segments (CATCH)		To clone a 36 kb jadomycin BGC	Jiang et al., 2015
		Combining CRISPR/Cas9 system with *in vitro* λ packaging system	*S. avermitilis* MA-4680	To clone 27.4 kb Tü3010 and 40.7 kb sisomicin BGC	Tao et al., 2019
	Reconstruction	Regulatory sequences cassettes	*S. albus* J1074	To activate the actinorhodin BGC.	Ji et al., 2018
		RedEx	*S. albus* J1074	To activate the spinosyn BGC and butenyl-spinosyn A production is 2.36 mg/l, spinosyn Jproduction is 7.34 mg/l	Song et al., 2020
	Chassis strain	Deletion phosphofructokinases (encoded by *pfk*)	*S. albus* J1074	To increase the precursor level of NADPH and heterologous expression of actinorhodin	Kallifidas et al., 2018
		Deletion genomic regions	*S. Chattanoogensis* L320	To delete 0.7 Mb non-essential genomic regions	Bu et al., 2019
Native host	Promoter	Identification strong promoters	*S. griseus*	To activate a PTM BGC.	Luo et al., 2015
		Characterization native or synthetic promoters and Ribosomal binding sites (RBSs)	*S. avermitilis*	To activate and overproduce the lycopene BGC.	Bai et al., 2015
		Identification a strong promoter *groESp*	*S. chattanoogensis* L10	To activate the natamycin BGC and to increase yield by 20%.	Wang K. et al., 2019
		Promoter engineering of the PAS-LuxR (*pimM*)	*Streptomyces*	To activate the polyene BGC.	Barreales et al., 2018
		CRISPR-Cas9 knock-in strategy	*Streptomyces*	To activate multiple BGCs of *Streptomyces* and to trigger the production of a pentangular polyketide.	Zhang et al., 2017
	Regulator	Expression of *bldA*	*S. coelicolor*	To activate the actinorhodin, undecylprodigiosin and methylenomycin BGCs.	Hackl and Bechthold, 2015)
		Overexpression of *slnR*	*S. albus*	To activate the salinomycin BGC.	Zhu et al., 2017)
		Heterologous expression of *papR2*	*S. lividans*	To activate the undecylprodigiosin BGC.	Krause et al., 2020)
		Expression of *gdmRIII*	*S. autolyticus*	To positively control the biosynthesis of geldanamycin.	Jiang et al., 2017
		Overexpression of *toyA*	*S. diastatochromogenes*	To activate the toyocamycin BGC and toyocamycin highest titer is 456.3 mg/l.	Xu et al., 2019
		Expression of *aveI*	*S. avermitilis*	To activate the melanin BGC.	Liu et al., 2019
	Ribosome and RNA polymerase	Overexpression of exogenous *rpsL* and *rpoB* genes containing beneficial mutations	Marine *Streptomycete*	To activate the piloquinone and homopiloquinone BGCs	Zhang Q. et al., 2020
		Mutation RNA polymerase: *rpoB* (H437Y)	*S. chattanoogensis* L10 (CGMCC 2644)	To activate the anthrachamycin BGC.	Li Z.Y. et al., 2019
		Mutation RNA polymerase:guanosine-tetraphosphate (ppGpp)	*S.sp.* SoC090715LN-16 S55-50-5	To identify/overproduce a novel isoindolinomycin.	Thong et al., 2018

## Heterologous Expression of Target BGCs

Heterologous expression is an efficient and established approach to unlock silent or cryptic gene clusters that have been identified by genome mining. Compared to expressions in native hosts, heterologous expression owns several advantages. (1) It can express BGCs whose native host is uncultivable or grows slowly under laboratory growth conditions; (2) heterologous host usually holds mature genetic manipulation tools; (3) the background information of the heterologous host is clearer (Xu and Wright, 2019). Generally, heterologous expression includes three steps-cloning of the target BGCs; engineering of the target BGCs; and transformation to the selected heterologous host. In this section, we will briefly update on the progress of heterologous expression from these three aspects.

### Cloning of Large BGCs

Traditional methods for cloning large BGCs generally employ genomic library constructed by cosmid, fosmid, BAC (Bacterial Artificial Chromosomal), and PAC (P1-derived Artificial Chromosome) vectors (Blodgett et al., 2005; Jones et al., 2013; Xu et al., 2016). In a recent research, 90 *Actinomycetes* NP BGCs have been successfully heterologous expressed, and about 83% of them were constructed via the cosmid/fosmid library method (Nah et al., 2017). However, these techniques are often time-consuming as well as laborious.

TAR (Transformation-Associated Recombination) cloning is a powerful and reliable system to directly clone large size BGCs (Orr-Weaver et al., 1981). The ends of the linearized TAR cloning vector contain specific homologous sequences of target BGCs as hooks to stimulate homologous recombination (Kouprina and Larionov, 2016, 2019). Bonet et al. (2015) reported the first case of heterologous expression of a natural product BGC from the marine *Streptomyces Salinispora* via the TAR-mediate pCAP01 vector. Later, Kang et al. (2016) built a mCRISTAR platform that combines CRISPR/Cas9 with TAR to simultaneously replace multiple promoters in the tetarimycin BGC. The system was further improved as mpCRISTAR (Multiple Plasmids-based CRISPR/Cas9 and TAR) by employing multiple plasmids, each harboring one or two unique guide RNAs. Based on mpCRISTAR, six or eight promoters can be simultaneously replaced with an efficiency of 68 and 32%, respectively (Kim et al., 2020).

Meanwhile, approaches based on site-specific recombinase systems have also been developed to directly clone BGCs. The integrase-mediated recombination (IR) system employs phage ΦBT1 *attP-attB-int* system to induce site-specific recombination (Du et al., 2015). Liu et al. (2009) described a versatile *E. coli-Streptomyces* shuttle vector system, pSBAC, employing the ΦBT1 IR system. Pyeon et al. further optimized the above system with additional restriction recognition sites on pSBAC to simplify the cloning procedure. They successfully cloned the 80 kb tautomycetin BGC and 60 kb pikromycin BGC for heterologous expression with the modified system (Pyeon et al., 2017). Zhang et al. (1998, 2000) developed a powerful Red/ET recombineering tool to assemble large DNA fragments using homologous recombination in *E. coli*. Later, Wang et al. (2018) upgraded this system by employing T4 polymerase to facilitate annealing between the linear target DNA and vector *in vitro*, and they termed the system as ExoCET. They used ExoCET to successfully cloned the intact 106 kb salinomycin BGC from *S. albus.*

Apart from *in vivo* cloning technologies, there are various *in vitro* cloning strategies. Gibson assembly has been well applied in multi-segment assembly *in vitro* (Gibson et al., 2009). For example, the 41 kb conglobatin BGC from *S. conglobatus* was cloned through Gibson assembly (Zhou et al., 2015). However, Gibson assembly is inefficient for large DNA fragments with high G + C content (Casini et al., 2014; Li et al., 2015). Therefore, Jiang et al. (2015) combined Gibson assembly with CRISPR-Cas9, termed as CATCH (Cas9-Assisted Targeting of CHromosome segments). They successfully cloned the 36 kb *jad* gene cluster from *S.venezuelae* and the 32 kb *ctc* gene cluster from *S. aureofaciens* into the p15A vector via CATCH. Similarly, Tao et al. (2019) illustrated an *in vitro* one-step targeted cloning approach combining CRISPR/Cas9 system with *in vitro* λ packaging system, and the pathways of Tü3010 (27.4 kb) and sisomicin (40.7 kb) were successfully cloned, respectively.

In summary, each strategy for cloning large BGCs has pros and cons. The methods of genomic library construction are random, but they are beneficial for metagenome-driven natural product discovery (Katz et al., 2016; Hover et al., 2018). The pSBAC is suitable for cloning large DNA fragments with specific restriction digestion sites, which are not generally available at both ends of target BGCs. As for tools based on homologous recombination, like TAR and Red/ET, both are commonly used in cloning large DNA fragments but may introduce some undesired recombination. Although CRISPR tools solve the limitation of insufficient restriction sites, it still faces the bottleneck of isolating targeted BGCs from the genomic DNA. With the increasing number of sequenced genomes, developing high-throughput cloning tools becomes imminent, such as combining current tools with automated platforms (Burger et al., 2020).

### Reconstruction of the Biosynthetic Pathways

It has been reported that the complexity of the regulatory network in host cells was a major challenge for metabolic engineering (Shao et al., 2013). Therefore, reconstruction and heterologous expression of the biosynthetic pathways can release them from the complex metabolic network. Nevertheless, BGCs controlled by promoters of different strengths increase the complexity of the reconstruction (Horbal et al., 2018). At present, the reconstruction process mainly includes: (1) gene substitution, (2) enzyme evolution, (3) promoter replacement, (4) transcriptional repressor knockout (Li L. et al., 2019). For example, Alberti et al. (2019) successfully activated the *scl* BGC by inactivating the transcriptional repressors via CRISPR/Cas9. AGOS (Artificial Gene Operon assembly System) is a plug and play method designed for the construction of artificial gene operons through Red/ET mediated recombination. Four gene operons of novobiocin BGCs were heterologously integrated into the genome of *S. coelicolor* M1146 via AGOS, leading to the production of novobiocin and novobiocin precursors (Basitta et al., 2017). Marín et al. cloned the synthetic genes encoding tyrosine ammonia lyase, 4-coumaroyl CoA ligase, chalcone synthase, chalcone isomerase and flavone synthase into a high copy number shuttle vector including a strong promoter *ermE^∗^p*. The final plasmid pAPI was transformed into the heterologous host *S. albus* and successfully produced apigenin at 0.08 mg/L (Marín et al., 2017). In another case, Song et al. (2020) refactored the spinosyn BGCs via RedEx to test whether the ethyl group at C-21 of spinosyn A can be replaced by butene group.

Among the above mentioned methods, promoter replacement is the most effective and well established method to activate silent BGCs, especially in *Streptomyces* (Luo et al., 2013). For example, Luo et al. identified strong promoters from *S. albus* J1074, whose strength is 200–1,300% the strength of the well-known strong promoter *ermE*^∗^p. They used a plug-and-play scaffold to successfully activate the silent PTM BGC of *S. griseus* in three widely used *Streptomyces* chassis strains (Luo et al., 2015). Ji et al. (2018) used synthetic regulatory sequences cassettes to successfully activate actinorhodin BGCs. In another case, the silent streptophenazine BGC in marine *Streptomyces S.sp* is non-transcriptional active in heterologous environment. After introducing four constitutive promoters (*ermE^∗^p/actIp/sp44/p21*) at different positions in the BGC, the production of streptophenazine was detected (Bauman et al., 2019).

All in all, thorough reconstruction of BGCs often leads to the activation of silent BGCs and the discovery of new NPs (Luo et al., 2016; Zhao et al., 2019). However, compared with *E. coli* and other model strains, the genetic manipulation tools in *Streptomyces* are still limited. Therefore, new methods are urgently needed and some new developments are well summarized in other reviews (Tan and Liu, 2017; Tao et al., 2018; Zhao Y. et al., 2020).

### Rational Engineering of Chassis Strains

*Streptomyces* are rich in inherently valuable secondary metabolites. Therefore, series of *Streptomyces* species have been developed as chassis to express heterologous BGCs, such as *S. coelicolor, S. lividans*, and *S. albus* (Ziermann and Betlach, 1999; Zhou et al., 2012; Myronovskyi et al., 2018). A suitable surrogate expression host should contain several essential features: (1) a variety of natural product precursors which are conducive to construct abundant complex molecules; (2) a simplified secondary metabolite background; (3) an efficient transportation system to transfer various bioactive compounds; (4) a known regulatory network; (5) a mature fermentation and upscaling process; (6) powerful genetic manipulation tools (Baltz, 2010, 2016; Myronovskyi and Luzhetskyy, 2019; Xu and Wright, 2019). Researchers did a comprehensive and detailed introduction of *Streptomyces* species used as heterologous hosts from 2010 to 2018 in another excellent review (Myronovskyi and Luzhetskyy, 2019).

The commonly used chassis engineering strategy is to reduce the background of secondary metabolism (Lee et al., 2019). Non-essential genomic regions and secondary metabolic genes mainly appearing in the end region of the chromosome. They are not stable and prone to chromosomal rearrangements, hence knocking out of them may generate clean background chassis strains. Ahmed et al. (2020) developed a set of *S. lividans* chassis strains. The *S. lividans* ΔYA11 was obtained by deleting 11 gene clusters (228.5 kb) and inserting two *attB* sites. Bu et al. (2019) rational constructed two genome-reduced *Streptomyces* chassis strains, the *S. chattanoogensis* L320 and L321, through multiple computational approaches and site-specific recombination systems, with non-essential genomic regions deletion of 1.3 and 0.7 Mb, respectively. Sometimes, the low yield of heterologous produced NPs may be due to insufficient precursors in the expression hosts. Therefore, increasing the supply of the precursors is a promising strategy. Borodina et al. (2008) rationally engineered the *S. coelicolor* A3(2) strain by deleting the phosphofructokinases (encode by *pfkA2*) gene, thus the precursor level of NADPH was increased and the production of actinorhodin and undecylprodigiosin were upregulated correspondingly. Dang et al. (2017) knocked out *pfk* in *S. hygroscopicus* ATCC 29253. The titer of rapamycin increased by 30.8% in the engineered strain. Kallifidas et al. (2018) successfully heterologous expressed actinorhodin in *S. albus*, and then further increased its yield by knocking out *pfk*_*SA*_.

Currently, a set of powerful bioinformatics approaches are developed to design chassis strains rationally (Ren et al., 2020). Meanwhile, the powerful genetic editing tool CRISPR has been applied in *Streptomyces* for genome engineering (Tong et al., 2019a,b; Zhang J. et al., 2020; Zhao Y.W. et al., 2020). These techniques are expected to accelerate the development of *Streptomyces* chassis strains.

Heterologous expression has numerous advantages, but some limitations still exist. (1) The size of SM-BGCs is highly variable (1–100 kb), and most are more than 10 kb. Currently, there is no certain method that is universal, large-size endurable, efficient, and high-throughput; (2) Because of the complicated metabolic networks of *Streptomyces*, clarify the interaction between the host strain and the heterologous BGCs is hard; (3) At present, *Streptomyces* chassis compatible with all NPs’ production has not been reported; (4) Current genetic manipulation tools of *Streptomyces* are not applicable in all species, thus more powerful and universal genetic tools are needed.

## *In situ* Activation of Target BGCs

The expression of NPs BGCs in *Streptomyces* is governed by a complex metabolic regulatory network. The production of antibiotics can be greatly enhanced by rewiring the regulatory network (Xia et al., 2020). Therefore, a better understanding and manipulation of the regulatory network in these silent BGCs could help to activate BGCs. In this section, we described different strategies to manipulate the regulatory modules in the native hosts for silent BGCs activation.

### Promoter Engineering

With regard to cluster activation, promoter elements are of indisputable importance as they are responsible for efficient transcription, which is the first stage of gene expression (Myronovskyi and Luzhetskyy, 2016). Promoter engineering employs a set of regulatory sequences with known functions, to release the following gene expression from the native complex regulations. Constitutive promoters commonly used to activate gene expression include: the promoter of the erythromycin resistance gene *ermE* of *S. erythraea*, *ermEp*1 and its derivatives (Bibb et al., 1985); the phage I19 originated promoter *SF14p* (Labes et al., 1997); and the engineered *kasOp*^∗^ promoter (Takano et al., 2005; Wang et al., 2013). Inducible promoters commonly used to activate gene expression include: the thiostrepton-inducible promoter *PtipA* (Holmes et al., 1993), the synthetic resorcinol-inducible and cumate-inducible promoters (Horbal et al., 2014), and the synthetic tetracycline-inducible promoter *tcp*830 (Rodríguez-García et al., 2005).

Since the strategy of knocking in promoters with multiple operon structure by homologous double-crossover recombination is often time-consuming and laborious, (Zhang et al., 2017) reported an effective CRISPR-Cas9 knock-in strategy in *Streptomyces*, and this one-step strategy was applied to activate multiple silent BGCs in five *Streptomyces* species. Similarly, Tong et al. (2015) also adopted the CRISPR-Cas9 system (deemed CRISPRi) to control the expression of target genes in *Actinomycetes*. The combination of the CRISPR system and promoter engineering approaches makes the experimental operation and procedure relatively simple and efficient.

At present, progress in activating silent BGCs in *Streptomyces* through comprehensive multi-promoter insertion is limited. Constructing promoters with a wide range of transcription initiation activities, transcription strength and robustness would promote effective activation of silent BGCs, and gene expression balance needs to be taken into considerations as well. In short, promoter-based gene expression activation methods still need improvement.

### Transcriptional Regulation Engineering

The biosynthesis of NPs in *Streptomyces* is regulated by precise regulatory systems, in which transcription factors (TFs) regulate the initiation level of transcription by binding to DNAs. In the era of synthetic biology, coordination of TFs regulations sometimes can activate silent BGCs, such as overexpression of positive regulatory genes or inactivation of negative regulatory genes in *Streptomyces*. For example, *bldA* of *S. coelicolor* can activate the expression of the antibiotics actinorhodin, undecylprodigiosin, and methylenomycin BGCs (Cuthbertson and Nodwell, 2013; Bhukya and Anand, 2017). Guo et al. (2018) used gene deletion, complementation, and overexpression to determine the MarR family transcriptional regulator (MFR) SAV4189 as an activator of avermectin biosynthesis in *S. avermitilis*. In addition to pathway-specific regulatory factors, global regulatory factors can also activate silent BGCs. For example, through genome sequencing analysis, gene knockout, and transcriptional analysis, the global regulator AdpA was found to be able to activate nikkomycin biosynthesis, and repress the biosynthesis of oviedomycin at the same time (Xu et al., 2017). Recently, Wang B. et al. (2019) reported a transcription factor decoy strategy for targeted activation of large silent polyketide synthase and non-ribosomal peptide synthetase, and discovered a novel oxazole family compound. Li et al. (2020) developed a base editing system that combines CRISPR-Cas9 with site-specific recombination to achieve successful genome editing in *Streptomyces* by programmed mutation of target genes, thereby achieving product biosynthesis (such as hygromycin B). Owing to their simplicity and ease of use, these strategies can be scaled up readily for the discovery of natural products in *Streptomyces*.

### Ribosome Engineering

Ribosome engineering is an approach to discover microbes with certain spontaneous mutations in their ribosome or RNA polymerase, through screening antibiotic-resistant mutants on Petri dishes (Zhu et al., 2019). It is suitable for gene activation and strain improvement, resulting in the identification of novel secondary metabolites, as well as the enhancement of enzyme production and tolerance to toxic chemicals (Ochi, 2017).

The *rpoB* gene (encoding the RNA polymerase β-subunit) can activate silent BGCs in various *Streptomyces* by rifampicin resistance mutations (up to 70 times at the transcription level). Analysis of the metabolite profile showed that *rpoB* mutants produced many metabolites undetectable in wild-type strains (Tanaka et al., 2013). Li Z.Y. et al. (2019) used site-directed mutagenesis to generate ten mutants with point mutations in the highly conserved region of *rpsL* (encoding the ribosomal protein S12) or *rpoB*. Among them, L10/*RpoB* (H437Y) activated anthrachamycin biosynthesis in *S. chattanoogensis* L10 (CGMCC 2644). Zhang et al. designed a TTO (Transcription–Translation in One) method using a plug-and-play plasmid system to directly overexpress exogenous *rpsL* (encoding ribosomal protein S12) and *rpoB* (encoding RNA polymerase β subunit) genes containing beneficial mutations. This method overcomes the false positive problem in the traditional ribosome engineering method and was successfully applied to activate the silent BGCs in three *Streptomyces* strains, thus discovering two aromatic polyketide antibiotics (Zhang Q. et al., 2020). Moreover, the ppGpp can interact with RNA polymerase and affect the production of antibiotics (Artsimovitch et al., 2004). It is suggested that RNA polymerases carrying specific *rif* mutations in the β-subunit can functionally mimic modification induced by binding of ppGpp (Xu et al., 2002). So, some studies showed that *rif* mutations could alter the gene expression patterns of ppGpp. Thong et al. (2016) screened mutants resistant to rifampicin and found an unknown metabolite.

At present, in addition to the conventional modification of ribosomes through mutagenesis, other ribosomal regulatory elements have also been engineered. Siu and Chen (2019) proposed a new class of riboregulators called toehold-gated gRNA (thgRNA) by integrating toehold riboswitches into sgRNA scaffolds and demonstrated their programmability for multiplexed regulation in *E. coli* with minimal cross-talks. In the future, this approach could also be tested in *Streptomyces* for gene expression regulation.

Promoter engineering can activate a single gene expression in BGCs, and it can also activate the full-length BGCs to produce the corresponding NPs. This method can be further developed for high-throughput activation of silent BGCs. Knockout of negative regulatory genes is one method to explore new NPs. However, in *Streptomyces*, the traditional gene knockout strategy is often completed by plasmid-mediated homologous recombination, which is usually time-consuming and laborious. Due to the differences in the source, structure and functions of BGCs, more attempts and innovations are needed to unlock the transcriptional regulation of BGCs.

## Discussion

At present, in addition to the methods mentioned in this review, the silent BGCs can also be activated by changing the culture conditions. Bode et al. (2002) defined it as one strain many compounds (OSMAC), that is, by adjusting the culture parameters of *Streptomyces*, such as medium composition, culture temperature, pH, aeration, and container type, to induce the expression of silent BGCs. Later, on the basis of OSMAC, other strategies were derived, such as the addition of low-concentration antibiotics, signal molecules and histone deacetylase inhibitors and other inducers (Seyedsayamdost, 2014), as well as co-cultivation strategies. In 2019, a review discussed the use of microbial culture techniques to expand the range of NPs available in the laboratory in recent years, mainly including methods such as adding physical scaffolds, adding small molecule elicitors, and co-cultivating with another microorganism (Tomm et al., 2019). Although these methods are relatively economical and simple, they are particularly suitable for *Streptomyces* species with incomplete genome information or genetic isolation defects.

Due to the high investment and low return rate of silent BGCs activation, the discovery of new NPs has entered a bottleneck. Through combining bioinformatics analysis with multi-omics data to explore the genomic data, insights to regulate and activate BGCs could be elicited. These methods can not only act alone to produce NPs, but can also be combined with each other. There is still an urgent requirement to develop better methods to activate silent BGCs. For example, structured data can be used to further elucidate the detailed mechanism, automation can help improving high-throughput capabilities, and AI can be employed to assist experiment design. Perhaps combining *in situ* activation with simulation analysis, heterologous expression and other strategies, more precise transcription activation could be achieved for silent BGCs exploration.

## Author Contributions

ZL, YZ, and CH: writing—original draft. YL: writing—review and editing and project administration. All authors contributed to the article and approved the submitted version.

## Conflict of Interest

The authors declare that the research was conducted in the absence of any commercial or financial relationships that could be construed as a potential conflict of interest.
